# Supplementary material for: Patterns of Intron Gain and Loss in Fungi
Source: PLoS Biol. 2004 Nov 30;2(12):e422. doi: 10.1371/journal.pbio.0020422 (PMC532390; doi:10.1371/journal.pbio.0020422)
Supplement: Table S1 — Also available at http://genes.mit.edu/NielsenEtAl/. (4.3 MB ZIP). [file pbio.0020422.st001.zip › NielsenEtAl/html/464.html]

AN6515.1.NCU01190.1.MG08069.1.FG07320.1


```
 CLUSTAL W (1.82) Multiple Sequence Alignments - Introns Inserted


Sequence 1: MG08069.1	333 aa
Sequence 2: FG07320.1	329 aa
Sequence 3: NCU01190.1	343 aa
Sequence 4: AN6515.1	345 aa
Alignment Length: 356 aa
Number Identitical Residues: 129 aa
Alignment Score (without introns) 7355


MG08069.1 	MASKT-DKIDKIIARLQQR~IAEGQF---YEAQQQTRVVAARYIKESSWGPATDILFNVS
NCU01190.1	MASKQDAKIQKIIARLQKR~IAEGQFEEQYEAGQETRLVAARYTKAENWPAAIDILSSVS
FG07320.1 	MAS----KVDRIVARLQAK~IEEGDY---YEAQQQTRVAASRYIKTSNWPAAIDILSSVS
AN6515.1  	MTS----RIDKTIARQREK2IASGAY---YEAHQQLRVIAARYIKQNNYDAAAEILAGGA
          	*:*    :::: :** : : * .* :   *** *: *: *:** * ..: .* :** . :

MG08069.1 	QSLLKAG----QGGSGGDLATLLV-DVYKQA---ELKPDSANKG-----~KLLTLLRLFA
NCU01190.1	QALLKAG----AGGAGGDLAILLV-DVYKQG---KVKPDAASRG-----~RLLTCLRLFE
FG07320.1 	QALLRAG----QGGSGGDLCAMLV-DVYKQA---ELKPDADSKG-----~RLLTCLRLFE
AN6515.1  	TALLRAGSQQGASASGGDLAIMLVTEVYVKAGWEIVGGDDDAEGRARKK1RLIELLREFP
          	 :**:**:... ..:****. :**::** :.. . :  *   .* : .. :*:  ** * 

MG08069.1 	PGEPTKKKFVTEMIG2WSSKFGEYPAGDPELHHVAGSLYAEE~HETSEAERHLILGTKDS
NCU01190.1	FEEPTRKKFVKEVFD2WSRKFGDYPAGDPELHHVVGTLAAEK~HETDEAEKHLVLGTKES
FG07320.1 	AGEPTRKKFVGEIIG2WSAKFGDYPAGDPELHHVAGSLYAEE~HDTYEAEKHLLLGTKDS
AN6515.1  	SEEPTRKRFIQEAIG~WSGRFGSVERGDPELHHAVGSVYAED1QEPYEAEKHLVLGTSES
          	  ***:*:*: * :. ** :**.   *******..*:: **. ::. ***:**:***.:*

MG08069.1 	AEALARMEYSWYKEDD-SHTAPLYAARAVLPYLLVGNVRAANACYRSFASALSADNQGMG
NCU01190.1	AEVLTRMEYEWYKDSEQPHTAALFAARAILPYLLLANVRAANTSYRAFVSALTAENSSLG
FG07320.1 	PEVLTKMEYTWYKESE-PHQAALFAGRAVLPYLLVGNVRAANTCYRLFTSSLSSDNPNLG
AN6515.1  	AETLAKLEYEWYTSDE-PHTAGIYAARAVIPFLLIGNLRSANKAFLIFTSRLSSANPSLG
          	.*.*:::** **...: .* * ::*.**::*:**:.*:*:** .:  *.* *:: * .:*

MG08069.1 	VQDVSTNSA-DLRVFPSLPLLNFLGMLLLAIQKGNPDAFRKLTMKYGPQIAEAAPAWQES
NCU01190.1	VQNVESSQGSDIRIFPSLPLLNLLGLLLLAVQKGTPDLFRQLKTKYASNLAELGGAWDGA
FG07320.1 	VQDVSSPAG-DIRIFPSLPLLNFLGFLLLAIQRGAPELYRALVSKYATQINEAG-SWAEA
AN6515.1  	VQDVSSATS-DVRVFPALPLLNFLNMLLLAIQRGTADLFKQLTAHYATHIKEAG-ILDDA
          	**:*.:  . *:*:**:*****:*.:****:*:* .: :: *  :*..:: * .     :

MG08069.1 	LESIAEMYFGIARPRQSNPLMDMMGSLFGGAGGAGG---AASRKPPARRVE-APAAEGLD
NCU01190.1	LEMIAEMYFGIQRPRQSNPLLDMMGSLFGGGGGGGGGAGAAGGRPPVRRVEAAPTAEGLD
FG07320.1 	LEMVGEMYFGLSKPRQSNPLMDMMSGFFGGGGGQPE---KPKRKQVTQRQD-APAAEGLD
AN6515.1  	LPQIGEQYFGINIPRQGNPLLDMMGSMLFGGGQDSGRR-SASGRGQSRKVEAPPSNMELD
          	*  :.* ***:  ***.***:***..:: *.*        .  :   :: ::.*:   **

MG08069.1 	
FG07320.1 	
NCU01190.1	
AN6515.1  	
          	
```
